# Radon Risk Communication through News Stories: A Multi-Perspective Approach

**DOI:** 10.3390/ijerph21101302

**Published:** 2024-09-29

**Authors:** María-Cruz Negreira-Rey, Jorge Vázquez-Herrero, Tania Forja-Pena

**Affiliations:** Department of Communication Sciences, Universidade de Santiago de Compostela, 15782 Santiago de Compostela, A Coruña, Spain; jorge.vazquez@usc.es (J.V.-H.); tania.forja.pena@usc.es (T.F.-P.)

**Keywords:** radon gas, news media, risk awareness, health communication, news coverage, risk perception, Spain

## Abstract

Radon is, after tobacco, the most frequent cause of lung cancer. Communicating about its risks with a didactic perspective so that citizens become aware and take action to avoid radon remains a challenge. This research is framed in Spain, where 17% of the territory exceeds the maximum radon limits allowed by the WHO, and aims to study the role and impact of the media in radon risk communication. A mixed methodological design is applied, combining content analysis of news published in the last two decades by local media in the most affected areas with interviews with journalists and a survey of citizens to provide a multi-perspective approach. The results show that, although news coverage of radon is becoming more frequent, it is a topic that fails to position itself on the agenda for effective communication. The media are the most frequent source of information on radon, although they are not considered by the public the most trustworthy one. News stories about radon focus mainly on health and research to inform about the radon levels to which citizens are exposed and the risks associated with cancer. Collaborative strategies between the media, organizations, and public administration seem key to advancing the fight against radon.

## 1. Introduction

Radon is a radioactive element that is naturally produced by the decay of uranium in the earth’s crust and escapes to the surface in the form of gas, although it can also be present in water. Radon gas is colourless and odourless, making it imperceptible to human senses. It is an important health risk agent in areas with a moderate or high incidence of the gas. Radon was declared a human carcinogen in 1988 by the International Agency for Research on Cancer [1]; it is considered an agent within group 1 as carcinogenic to humans and has been recognized as the second most frequent cause of lung cancer after tobacco [2].

In the 1980s, studies on the effects of radon on health and its presence began to intensify, and at the same time the first steps were taken in the regulation for prevention. In Spain, the Nuclear Safety Council (CSN) and Enusa initiated in 1991 the Marna Project to measure the levels of natural gamma radiation in the country [3]. Later, the CSN also published the Predictive Map of Radon Exposure in Spain [4] and the Cartography of Radon Potential in Spain [5]. The results revealed that 17% of the national territory is a priority action area with radon concentration above 300 bq/m^3^ [6], the maximum limit set by the CSN and World Health Organization (WHO), although the objective is not to exceed 100 bq/m^3^ [4,7]. By regions or autonomous communities, the percentages of the territory exceeding this limit are as follows [6]: Galicia (70%), Extremadura (47%), Madrid (36%), Castilla y León (19%), Canarias (19%), Cataluña (16%), Asturias (12%), Ceuta (11%), Castilla-La Mancha (10%), Andalucía (8%), Navarra (6%), Aragón (2%), País Vasco (2%), and Murcia (1%).

Radon exposure and its risks are associated with indoor spaces where people live, especially workplaces and homes. There, radon emanates from natural sources, building materials, artificial sources, or even home appliances [8]. The radon concentration is influenced by the characteristics of the soil, the houses’ insulation quality, or the elevation—basements and first floors tend to be the most affected because as the height of the floors increases, the concentration of the gas falls [9], although the stack effect can inverse this [10]. 

Older homes or those with deficiencies in their construction are also the most vulnerable to radon, due to poorer insulation conditions [11]. Therefore, and although data that take into account this variable are not available, it can be expected that the socioeconomic level for access or renovation of dwellings may also be a risk factor in relation to radon exposure. Based on CSN [6] measurements, performed on ground and first floors of buildings, Ministerio de Sanidad [9] has provided indoor radon exposure prevalence data by region, correcting the data for house height. This correction is important for weighting the risk in regions such as Madrid, where there is a high percentage of vertical buildings in urban areas [9].

The regulatory framework is determined by Directive 59/2013 Euratom, which obliges member states to define a national radon plan. In Spain, the directive was partially altered with Royal Decree 732/2019, which updated the Technical Building Code to require, in new or remodelled buildings located in the highest risk municipalities, that the concentration of the gas does not exceed 300 bq/m^3^ [11]. Three years later, Royal Decree 1029/2022 was approved to establish standards for the health protection of the population and workers against the dangers of ionizing radiation [12]. Finally, in 2024 the Council of Ministers approved the National Plan against Radon [11], which establishes five strategic objectives, one of them to raise awareness among the public, professionals, and administrations.

The final objective of the National Plan is key to advancing awareness and the prevention of health risks. In 2017, 838 deaths were attributed to radon exposure in Spain, mainly attributable to smokers and ex-smokers [9]. Communication is essential for the public to know what radon is, the level of exposure to which they are subjected, the associated risks, and the measures they can take to prevent it. The media also play a role in risk communication, since information habits and media literacy are important for the health literacy of citizens [13,14].

This research is based on the above premise, framed in the Spanish context, to study the news coverage and risk communication about radon gas in the journalistic media, with a mixed methodological design to study the professional and citizen perspective. The research responds to the following objectives.

O1. To study the news coverage on radon gas in the local media in areas with the highest incidence of the gas in Spain by analysing published news to determine its temporal evolution, subject matter and scope, sources of information, or degree of proximity.

O2. To observe how the news story is constructed to communicate the health risk associated with radon gas from journalistic media, analysing the description and explanation of the risk, the main actors appearing in the news, and the conditions of journalists to report on radon and health.

O3. To discover the perception of the Spanish population in relation to the information they receive about radon gas and its risks through journalistic media.

## 2. Background

### 2.1. Risk and Health Communication about Radon Gas

Communication strategies about the health risks associated with radon gas are linked to the study of the public perceptions of the hazard [15]. Communicating effectively about the risks is challenging: the gas has a natural origin and it is no visible for the human eye; people perceive the home as a safe place, which may lose property value if the hazard is recognized; it is difficult to see the direct health impact or to associate cancer deaths with radon exposure; and, in addition, it can generate uncertainty if the messages being disseminated are contradictory [16,17,18,19].

The WHO [7] proposes focusing communication strategies on the risk of lung cancer, emphasizing the greater danger to smokers, and comparing radon with other causes. However, different investigations in various geographical and social contexts show that the level of knowledge and information that citizens receive about radon is generally low, whether they live in areas with higher or lower incidences or are more or less concerned about their health [15,20]. When information is available, it may not reach the target population correctly or it may be difficult to understand [15,21]. Thus, national and regional strategies aimed at radon risk communication are essential [22], as studies conducted in Bulgaria and Germany have shown [23,24], also remarking that communication on radon should be sustained over time, repeating messages and campaigns to keep the topic on the agenda.

The studies and communication plans developed over the last three decades have led to common conclusions and recommendations. For citizens to feel that radon is something they can and should act against, it is proposed that it should no longer be described as a “natural radioactive gas” but rather as “indoor air pollution” or a “harmful substance in the indoor air” [20,23]. The importance of combining the technical and informative dimension—the most frequent and preferred by scientists—with the emotional and social narrative in communication is also emphasized, seeking balance with concise, clear, credible, and persuasive messages [19,25,26].

This radon narrative is constructed by personalizing the risk with testimonials, showing radon as a serious problem, relating it to cases of people suffering from lung cancer, comparing its risks with other tangible and relevant ones, insisting on the need to test all homes, alluding to family and child care, explaining the methods and costs of measurement and evacuation measures, being didactic with solutions, and debunking myths about the gas [19,20,25,27,28].

Radon risk communication should seek the presence and cooperation of expert scientists, organizations, and communicators, with the presence and credibility of national and local authorities being important [15,23]. The use of the widest possible variety of mass media—newspapers, television, radio, digital media, social media, and apps—is recommended in coherence with the communication strategy and the audience [15,26]. Proximity is also a relevant factor in risk perception and communication, as it strengthens trust in institutions and the willingness to take preventive and remedial action [29].

### 2.2. The Role of the News Media in Radon Risk Communication

Social, cultural, political, and economic factors must be taken into account in the communication and perception of the risk associated with radon [30]. While scientists have a scientific–technical function and political managers have a bureaucratic–managerial one, the media must be social amplifiers of risk to generate an impact on the perception of citizens [30]. In this research, we study the role of the media as actors in risk communication through the content of their media coverage and citizen perceptions [31,32].

The role of the news media in communication strategies is essential since, due to their reach, credibility, and reliability, they are the most important information channels for communicating risks to the general population [33]. However, news coverage of certain risks may depend on their “newsworthiness” according to classical news values and, in turn, be influenced by the social context—the social perception of risk—and political angle—conditions and dependencies on power [31,34]. It is difficult to know which factors—such as personal stories or local information—are most influential in making radon a newsworthy topic [35]. Sustaining media coverage of radon over time also requires that sources act as constant providers of information, for which the media must be responsive [35].

Research on radon coverage in the media reveals a relevant change in recent decades. In the 1980s, there was a predominance of official government sources and very limited presence of news stories mentioning the health risks and the relationship of radon to cancer [34]. However, the results of a study that analysed the news in the period 2003 to 2014 [16] reveal that, in recent years, there has been an increase in mentions of cancer, health effects, and the promotion of making measurements in homes, which respond, in part, to changes in government regulation and research results. However, the most recent studies continue to find poor news coverage and low media involvement in radon risk communication [36].

## 3. Materials and Methods

The methodological design combines quantitative and qualitative methods, which have been conducted in several phases of research to meet the objectives formulated.

### 3.1. News Media Coverage

First, the research analyses the news coverage on radon gas (O1, O2) in 23 local digital media—native and legacy—, of generalist themes that operate in the most affected Spanish provinces, located in the regions of Andalucía, Asturias, Castilla-La Mancha, Castilla y León, Comunidad de Madrid, Galicia, and Extremadura [6]. 

The media outlets were selected by searching for the reference and longest running legacy and digital native titles by province and region. From the sample of media in Table S1, news published in the period 2002–2022 containing the word “radon” were collected through a search in the newspaper archives of their digital editions. A total of 1049 news items were retrieved, from which a random sample was selected for the analysis, keeping a confidence level of 95% and a margin of error of 5%, which resulted in an analysis sample of 579 items.

A quantitative and qualitative analysis of the news content was carried out, focused on the headlines and the main text of the news items. The analysis considered the following variables: identification data of each item; main topic (radon or others); news values; thematic focus; magnitudes of risk; sources of information; and geographical scope (Table S2).

### 3.2. Interviews with Journalists

To complete the analysis of how news stories on the health risks associated with radon are constructed, we sought to understand the conditions under which journalists produce such content in the local media (O2). To this end, we opted to design a questionnaire addressed, in the first place, to managers, editors-in-chief, and journalists from the health, environment, and society sections—as these are the most common sections for news on radon—of the media outlets in the sample. With the aim of expanding the sample of professionals who could contribute, 36 journalists’ associations in the regions studied were contacted to distribute the questionnaire among their members. The questionnaire was distributed through Microsoft Forms (Office 365) for self-administration and consisted of 28 questions, arranged in the following sections: (a) identification data; (b) risk communication and media; (c) approach to the object of study (knowledge about radon gas); (d) communication about radon gas.

### 3.3. Public Perception

To determine the perception of the Spanish population on information about radon gas (O3), a survey was designed for the population over 18 years of age by using a self-administered online questionnaire (CAWI) with multiple choice questions, dichotomous response, or a Likert scale 1–7. The questionnaire was organised in the following sections: (a) sociodemographics; (b) news interest and news use; (c) information on radon: channels and sources; (d) communication actions on radon; (e) trust in institutions and groups. A proportional multistage sampling was performed at the level of regions and random selection of households, habitat of residence, gender, and age of the subject. The confidence level was set at 95.5% (P = Q as the most unfavourable case) and the maximum error allowed for the sample as a whole was ±2.18%. The survey was conducted by Sondaxe, a company specializing in opinion polls, with a target of 1500 responses. After applying objective criteria for invalidity of responses, the sample consisted of 1442 participants (Table 1), who responded between March and June 2023. The data were examined using descriptive statistical analysis. The first step was an analysis of means and frequencies, followed by ANOVA test to determine statistically significant differences in information consumption preferences between sociodemographic groups (age, gender, level of education, radon incidence by region). IBM SPSS Statistics 29.0.2 software was used for this task.

## 4. Results

### 4.1. Radon Gas News Coverage

The content analysis of the news (n_1_ = 579) allowed us to make a general characterization of the news coverage on radon in the local media of the Spanish regions most affected by the gas (O1).

**Figure 1:**
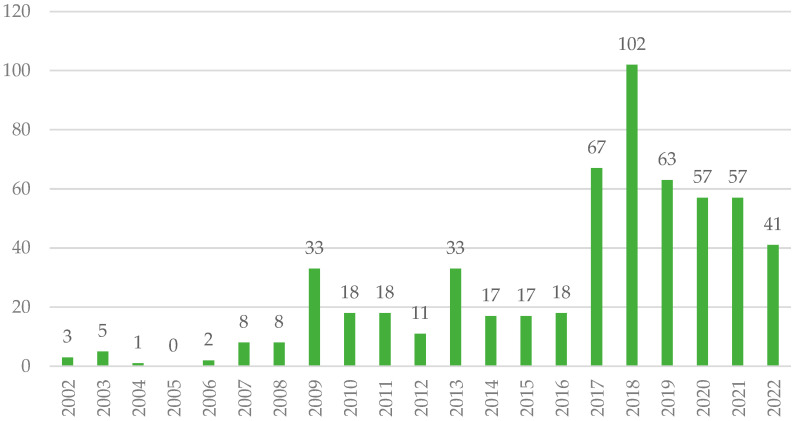
Number of news items published about radon per year in the analysis period (n_1_ = 579). Source: own elaboration.

The first data that we observed are the volume of publication of the news analysed per year (Figure 1). In these 20 years, we identified an increasing evolution in the number of news items published on radon—particularly remarkable since 2017.

Analysing the thematic focus in general, it is observed that in 83.7% of the news items, radon was mentioned in relation to health, in 46.1% to research, in 39.5% to housing and urban planning, in 28.4% to politics, in 15.2% to the environment, and in 9.4% to other topics. It is common for two or more thematic approaches to coexist in one news item.

Radon gas was the main topic in 48.7% of the news items analysed. In general, the information is related to maps of gas incidence by geographical areas, measurements, prevention actions—solutions in homes, public buildings, etc.—or with research projects, their results and dissemination actions.

Among the news items in which radon was a secondary topic, those related to cancer stand out. In these cases, radon appeared as a risk factor associated with the development of this disease, mainly lung cancer. In the rest of the news, we found a series of frequent topics: political issues, in relation to the holding of municipal plenary sessions to discuss plans to measure and implement solutions against radon, or on subsidies to apply meters and gas evacuation systems in buildings; construction and urban planning policy, in relation to measurements, renovations, and solutions in public and private buildings; research projects and outreach activities on radon gas, its incidence, or health risks; occupational safety and risks in the workplace; and the environment and natural surroundings, in relation to the presence of the gas in caves, hot springs, mines, or its expulsion in earthquakes, volcanic eruptions, or geysers.

Regarding news values, the most frequent were novelty (77.0%) and relevance (72.9%). Radon is not a topic that receives continuous attention from the media, but it manages to position itself on the agenda when something new happens and is considered relevant. As already mentioned, the most recurrent news on radon report new plans or measurement results by geographical areas, new research results in relation to health or other factors, the approval of new actions, political measures, or economic aid to take solutions and reduce exposure to radon. In general, the novelty is linked to the relevance of the risk posed to human health by exposure to radon gas, the importance of knowing about it and being able to prevent it. The news value of the conflict was much less frequent (12.8%) and appeared in news items, generally at the municipal level, in which political discussions about radon or protests by unions and workers due to exposure to dangerous levels of radon in their workplaces were reported.

Regarding the information sources cited in the news (Table 2), most of the pieces only cited one source (61.0%), with those citing two (20.7%) or three (10.0%) being less frequent. According to the type of source (Table 3), the most frequently cited were research institutions or organizations (33.1%). Among these, the presence of the World Health Organization stands out as the reference institution that sets radon concentration levels that pose a health risk, or the Nuclear Safety Council, which, in Spain, is the referent in the publication of national mappings of the presence of the gas. Also relevant was the presence of research groups specialized in radon or radioactivity belonging to Spanish universities, such as the Galician Radon Laboratory (University of Santiago de Compostela, USC), the Radiation Analysis Laboratory (USC), the Nuclear Radiation Experimental Group (University of Extremadura), the Environmental Radioactivity Laboratory (University of Cantabria), or the Ionizing Radiation Laboratory (University of Salamanca). Other research centres or entities related to health—such as the Spanish Society of Oncology, the Spanish Society of Pneumology and Thoracic Surgery, or the International Agency for Research on Cancer—, geology and the study of the natural environment—such as the Institute for Geoenvironmental Health, the National Geographic Institute, or the Geological and Mining Institute of Spain—, and other reference institutes such as the Spanish National Research Council (CSIC) were also frequently cited.

Sources belonging to the public administration were the second most cited type (23.6%). Among these, the most frequent were those belonging to local government bodies: city councils, regional government departments—in health, urban planning, infrastructure, etc.—and provincial councils. Sources from the national government and some ministries appeared with less weight. The third type of sources most cited were experts (19.1%), among whom we found, in general, researchers from the aforementioned research groups and institutions (mainly those specialized in radon) and reference professionals in the field of health (mainly oncology and pneumology), geological and natural environment studies or with experience in the design of solutions against radon—especially measurement and evacuation of buildings. Civil associations (8.2%)—workers’ unions, professional associations, cancer and environmental associations; companies (6.4%) specialized in gas measurement and disposal; political parties (5.6%)usually as part of the political opposition to the aforementioned government bodies; and citizens (3.0%)—appeared with less weight. According to the degree of proximity, 31.8% of the sources cited were regional, 26.5% were national, 25.7% were local, and 13.7% were international.

Regarding the geographic scope or degree of proximity of the news (Table 4), it was observed that news stories on radon gas were constructed from local and closer to the citizens spaces . This was also evident in the thematic sections in which the media classified the news about radon (Table 5), whereby the local section was the most frequent (63.3%), followed by society (14.0%) and health (5.4%).

In addition to building stories about radon from proximity, how the risks associated with exposure to the gas are related and explained is important. Therefore, a qualitative analysis of the reporting on the risk magnitude in the news has been carried out. It is noted that, although the news presented the latest data on radon, they often included a contextualization explaining what it is, how it is produced naturally in the subsoil, the geological characteristics of the most affected areas, or its presence in the air and water. The different risk levels defined by the WHO in relation to gas concentration were also explained, as well as the European regulatory framework and the slow transposition in Spain. As we have seen, radon appears very frequently related to health, since in the news it is also frequently explained that exposure to the gas is an important risk factor in the development of cancer, especially lung cancer. In the description of the risk, citizens were also frequently informed about possible solutions: daily ventilation of indoor spaces was emphasized as the most affordable and effective measure, but also regarding options for measuring the gas, installing evacuation systems, or about policies and measures to support building renovations.

### 4.2. Journalists Reporting on Radon Gas

During the distribution of the questionnaire, five responses were obtained between May and October 2023. The anonymity of the journalists is preserved, but we can point out that all of them are professionals in regional and provincial media outlets with different positions: a director, two editors-in-chief, an editor of health and biosanitary research, and a reporter.

All the journalists surveyed considered the level of radon incidence in their region to be high or medium, and four agreed or strongly agreed that it is a risk to public health. All of them also agreed that the public is not well informed about radon gas, although three of them pointed out that the public does not demand information about it either.

When assessing the performance of the media in general, all journalists stated that they are not communicating effectively about the dangers of radon. When evaluating the news coverage of the media in which they work, one considered it appropriate, another one considered it sufficient, and three others considered it null. They attributed this negative assessment to the fact that radon, as a news topic, does not occupy an important or continuous place on the media agenda, therefore it is not given notable coverage. One of them related this lack of relevance in the agenda with the perception of risk, because “although radon has harmful effects on health, its incidence is not immediate and therefore it is perceived as a low risk”. Another one pointed out the difficulty in finding in the local environment “outstanding research, local specialists who campaign on the subject […], as if it were a secondary problem, not important, to which not much attention is paid”. On the other hand, the director of one of the media outlets analysed recognized that there may be “an important business and university pressure to avoid bad information”, while a reporter believed that there could be “economic interests behind the companies that work with radon”.

Journalists believed that the knowledge of the public and the information they receive about radon gas could be improved by producing more reports on the subject, giving a voice to experts in the field, taking advantage of the celebration of European Radon Day on November 7 to place the issue on the agenda and seek impact on social networks, and, in general, by expanding the information available.

Regarding their media coverage, three of the five journalists interviewed stated that they always or sometimes produce information on their own initiative, based on press releases from research institutions or the public administration. Only two pointed to communications from companies in the sector as a starting point for news coverage. In relation to sources, journalists identified experts, academics, and research institutions, as well as civil associations as the most frequently cited or consulted. Political parties, public administration, companies, and citizens were the least frequently mentioned. The informative focus they identified as the most common in the news about radon was health and prevention, followed by housing and urban planning, research, and environment. None of them identified a policy and regulation-oriented focus in radon news.

The journalists interviewed unanimously agreed in identifying cancer, specifically lung cancer, as the risk associated with radon that was most widely reported. They recognized that media outlets were responsible for the news they disseminate, but that it should be the public administration and the scientific community who assume responsibility for alerting the public to the dangers associated with radon. From journalism and the media, professionals agreed that risk communication should be managed with rigor, responsibility, and objectivity. They said that it is necessary to give “clear and explanatory information, being very didactic about the risks”, telling the news “without alarmism or looking for easy headlines”. The journalists interviewed also recognised that it is important to “follow ethical principles, follow the recommendations of professional associations, have a person in charge of coordinating this type of information in the newsroom and have reliable official sources available, not interested ones”. It can be concluded that “the media are the most effective vehicle for transmitting messages as important as risk communication, because citizens rarely go to direct sources, but receive recommendations directly from the media”. In addition, one of them remarked that “the media must be rigorous when filtering what is really important; be specific and local”.

### 4.3. Public Perceptions of Radon Risk and Communication

The Spanish population showed a medium-high interest in being informed about what is happening in their environment and in the world (M (mean) = 5.4 out of 7), with 25.9% saying they were “extremely interested”. This interest was higher the older the individual (*p* < 0.001) and increased along with level of education (*p* < 0.001).

Although disaffection and distrust of the media is a global phenomenon, the Spanish population manifested active news avoidance at moderate levels (3.1 out of 7), with 26.0% expressing avoidance (5–7 on the scale). There were statistically significant differences by age, with news avoidance being higher the younger the individual (*p* < 0.001).

The main channels consulted (in reference to the week prior to conducting the survey) were, in order: television (73.2%), digital media (45.6%), radio (37.2%), Facebook (30.7%), written press (28.8%), YouTube (28.8%), WhatsApp (26.4%), Instagram (24.1%), and Twitter (21.9%). Regarding the media they consult for information daily, the most frequent was television, followed by digital media and social networks, followed by radio, instant messaging apps, and press.

When asked through which channels they had received information about radon at some point (Table 6), the answers were generally coincident with the most used media, taking into account that 46.5% said they received information through media outlets: 30.1% indicated having received information about radon through television, followed by digital media (20.1%), press (12.9%), and radio (11.5%). A total of 19.3% indicated that they received information about radon through social networks and instant messaging apps, including YouTube, Facebook, WhatsApp, and Instagram.

Statistically significant differences by gender and age were identified. More men indicated having received information about radon through mass media (*p* < 0.001); through networks, it was more frequent among 18–29-year-olds (*p* < 0.001). In communities with a high incidence, no statistically significant differences were detected with respect to other regions.

Regarding the sources cited in the news content on radon (Table 7), the government was the most frequently recalled source (24.7%), followed by NGOs (16.7%), the Spanish Nuclear Safety Council (14.9%), and political parties (8.1%), with the important limitation that 45.3% did not remember the origin of the information. A tendency was identified in male citizens, who remembered more frequently that the information came from the government or NGOs. And a statistically significant difference was noted by age: the younger the person, the more they indicated political parties as the source, both in the 18–29 and 30–44 age groups (*p* < 0.01). No statistically significant differences were detected in the communities with high incidence.

We complemented this approximation to the informative perception of the population by inquiring into the information received through communication actions implemented by different institutions or groups (Table 8). Only 8.7% knew that specific radon risk communication activities had been carried out in the country. And the best support for transmitting preventive information about this gas, according to citizens, were media outlets (for 68.4%) and social networks (46.4%). The main finding is that 58.2% considered that they have not been informed about radon. The results show that the main source of information on radon is media outlets (19.5% of those surveyed indicated having received information from them). The European Union, scientific organizations, family and friends, and the workplace were the next most important sources of information about radon. Below 8% were the state, regional, and municipal governments, followed by other groups and institutions at levels below 5% and representing exceptional cases. However, these data should be evaluated taking into account that they correspond to the perceptions of citizens.

Through the ANOVA test, multiple variables were identified in which there is a statistically significant difference by gender or age, although not by the level of radon incidence by community. Statistically significant differences were also identified in some of the variables by gender (higher in men) and by age (higher in the 18–29 age group in general), although these data are not of particular interest for this study.

With respect to trust in the aforementioned institutions and groups, scientific organizations (5.2 out of 7) and the Nuclear Safety Council (4.9) stand out in first place. In second place are the faculty (4.8) and the school (4.7), as well as the living environment—friends and family (4.66). This was followed by the workplace (4.5), the European Union (4.4), and media outlets (4.2). At the lowest level were regional (4.0), municipal (3.9), and state (3.9) government bodies.

**Figure 2:**
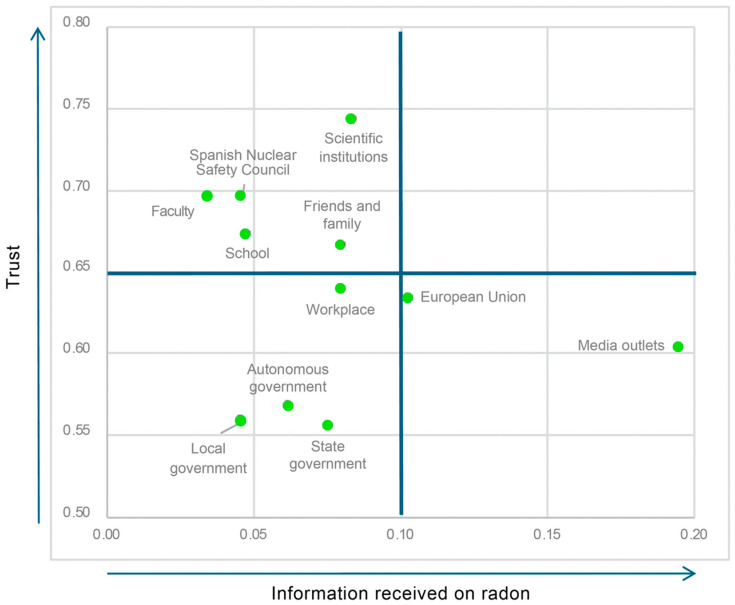
Quadrant graph based on the relationship between information received about radon and trust in the institutions or groups from which it originates. Source: own elaboration.

These results reflect this dispersion between the level of information and trust that citizens have in the actors involved in radon risk communication. To explore this issue, and as a tool for analysis and design for future communication actions, we constructed a graph based on quadrants (Figure 2) in which the different institutions or groups are placed according to their impact on the public (information received, X axis) and the trust of citizens in them (Y axis). Thus, we can see that the first quadrant (top right) is blank and represents those actors who would be recognized as sources of information on radon and who are also trusted by the public. The second quadrant (top left) represents the actors that generate trust but are rarely identified as sources of information. In the third quadrant (lower left), we find actors who are neither valued as trustworthy nor as frequent sources of information on radon. The fourth quadrant presents those actors who are identified as sources, although trust in them is not high. The main reading that can be drawn is that the media are the most frequent channel of information, while scientific organizations, the Spanish Nuclear Safety Council and living environments (family, education, and work) are the ones that generate the most trust but are not perceived as present in the media.

## 5. Discussion

This analysis has allowed us to respond to the proposed objectives and offer an overview of risk communication about radon gas in the news media in Spain, incorporating the perspective of journalists and citizens. In relation to the news coverage on radon in the Spanish local media (O1), it is observed that, even though this is not a leading or continuously covered topic on the agenda, the volume of news has been increasing since 2017. This was the year when the Nuclear Safety Council published the Cartography of Radon Potential in Spain, which remains the reference document for the incidence of the gas in the country. In these years, the national regulatory framework for action against radon was being built up, a factor that could be related to the increase in the intensity of information on the subject. Since 2016, European Directives 51/2013 Euratom—to establish health protection referring to radioactive substances in water for human consumption—and 59/2013 Euratom—which urges member states to design comprehensive national action plans against radon—were introduced. The former was incorporated in Spain with Royal Decree 314/2016 to regulate the exploitation and commercialization of bottled water for consumption. In 2019, the national government updated the Technical Building Code and in 2022 Royal Decree 1029/2022 was approved, which laid the foundations for the 2024 National Plan against Radon, for the protection of health against the risks arising from exposure to ionizing radiation, which is related to the implementation of the Strategic Plan for Health and Environment approved in 2021.

The analysis of news coverage on radon gas evidences that, in social communication about the radon-related risk, the media fulfil their role as intermediaries [30], amplifying the updates that have occurred in this period, such as the publication of the national radon maps of the Spanish Nuclear Safety Council [6], the intensification of new research results, and the progressive transposition of European regulations.

The news story is dominated by health and research issues, which is also reflected in the most cited sources—scientific institutions and research bodies such as the WHO or the CSN. Policies and governmental actors remain in the background, although they are also relevant at the local level because they are the ones who regulate and manage the solutions that most directly affect citizens. As in previous research [16], in the Spanish case it is also observed that media coverage has been oriented towards health and the prevention of risks associated with radon.

The narrative that is built about radon from the journalistic media (O2) is marked by an informative and technical tone, rather than by the social or emotional dimension [26]. Although expert, technical, and administrative voices predominate, data on radon incidence or those related to cancer are usually contextualized with explanations of the nature of the gas, prevention measures and solutions, or steps in regulation. Although in a very limited way, an explanatory and even didactic dimension is evident in some of the news, which is a key factor for citizens to feel empowered to make measurements in their homes and to implement solutions [23,25].

In addition, the news stories analysed have a marked local character—reflected in the sources and geographic spaces of the information—which helps citizens feel more connected and aware of the risk, in addition to strengthening trust in local institutions and actors [29]. The news stories about radon gas were told from local spaces because this area of proximity is more relevant for citizens. In addition to knowing about the presence of the gas, the risks or the regulatory framework in the more general context, citizens need to know what levels of radon they are exposed to in their homes, places of study or work, as well as the risks this poses to their health or the solutions and measures they can take. That is why we see that the proximity is in the thematic focus of the news or the sources cited.

However, the journalists’ perspective (O2) is not very positive. They recognize that news media are not communicating effectively about radon and its risks, as it is difficult to place the issue on the agenda and find expert sources in the local environment. Therefore, an alliance between the scientific, political, and communication sectors [15] seems necessary to build a reciprocal relationship in which sources would be proactive and the media receptive to their news [35]. Nevertheless, journalists believe that their role is fundamental in risk communication, which must be carried out with rigor and ethics, while at the same time being clear, explanatory, and understandable to citizens. As a limitation of the study, the final number of responses obtained is admittedly low, although they allow for an approximation of radon coverage from a professional and media management perspective, which complements the results of the content analysis.

From the perspective of citizens (O3), it is found that they are generally poorly informed about radon—58.2% stated that they have not received any communication about the gas—a problem that has also been found in other contexts [20]. Despite this, media outlets are the main channel through which citizens claim to receive information about radon (46.5%), the most frequent being traditional media (television, written press, radio). However, scientific institutions and groups are the actors they perceive as the most reliable, followed by the family and work environment.

The role of the journalistic media must be supported, due to their ability to disseminate wide-reaching, reliable, explanatory, and contextualised information from an unbiased position. However, although the proximity of local media continues to be a strength [37], the media are also victims of a progressive loss of trust and a growing news avoidance by citizens [38]. On the other hand, the institutions that are more trusted should take advantage of this potential to design more effective communication strategies through different media, including journalistic outlets or their own profiles on social networks. Those platforms, due to their wide population penetration and widespread use, have great potential for health and risk communication [39], although some studies reveal that radon is a topic that is seldom addressed on social media [40].

## 6. Conclusions

In the context we described above, it is necessary to assess what role the different actors should or want to play in radon risk communication. News media remain an ideal channel for information dissemination, awareness raising, and education about the risks and solutions in the local areas most affected by radon [13]. However, we cannot ignore the problems of trust and news avoidance that journalism suffers from today [38]. Revaluing the professional role of journalists with quality information will be essential, for which direct and continuous collaboration with expert sources in the scientific and health sector is required.

However, public administrations and government bodies must take responsibility for regulating, managing solutions, and articulating plans to support citizens. They must also integrate communication strategies that combine the national and local dimension, take into account the different audiences and their needs, and adapt messages and channels to reach them effectively. In Spain, the recent National Radon Plan [11] proposes a strategic axis focused on communication and awareness. Actions are planned to learn about the public perception of radon and its risks, analyse existing communication, and develop outreach campaigns aimed at specific publics. Future research will need to address the study of these strategies, while advancing regulation and research, to verify the real impact of action taken against radon on the public.

## Figures and Tables

**Table 1:** Sociodemographic data of the survey sample (n_2_ = 1442).

		%
Age (years)	18–29	13.6
	30–44	31.4
	45–64	48.3
	≥65	6.7
Gender	Female	51.2
	Male	48.7
	Other	0.1

Source: own elaboration.

**Table 2:** Frequency of news items according to the number of sources cited (n_1_ = 579).

No. of Cited Sources	Frequency	
0	8	1.4%
1	353	61.0%
2	120	20.7%
3	58	10.0%
4	17	2.9%
5	14	2.4%
6	5	0.9%
7	2	0.3%
8	2	0.3%

Source: own elaboration.

**Table 3:** Type of sources in the news and frequency of citation (n_1_ = 579).

Type of Source	Frequency	
Research institutions	319	33.1%
Public administration	228	23.6%
Experts	184	19.1%
Civil associations	79	8.2%
Companies	62	6.4%
Political parties	54	5.6%
Citizens	29	3.0%
Others	10	1.0%

Source: own elaboration. The sum of the “frequency” is greater than the number of news items analysed because each news piece can have several sources cited, of different types.

**Table 4:** Geographical scope of the information (n_1_ = 579).

Degree of Proximity	Frequency	
Local	300	51.8%
Regional	178	30.7%
National	154	26.6%
International	31	5.4%

Source: own elaboration. The sum of the “frequency” is greater than the number of news items analysed because a single news piece may combine two or more geographical approaches.

**Table 5:** Ranking of the news published on radon in the thematic sections of the media (n_1_ = 579).

Section	Frequency	
Local	365	63.0%
Society	81	14.0%
Health	31	5.4%
Environment	18	3.1%
Politics	16	2.8%
Science/Research	11	1.9%
Breaking News/Live	10	1.7%
Spain/National	10	1.7%
Technology	8	1.4%
Economy	6	1.0%
Others	6	1.0%
Specials/Features	5	0.9%
University	4	0.7%
International	3	0.5%
Education	2	0.3%
Trends	2	0.3%
Travel	1	0.2%

Source: own elaboration.

**Table 6:** Channels through which information on radon had been received at some time (n_2_ = 1442).

Channel	Frequency	
*Media outlets (any category)*	671	46.5%
Television	434	30.1%
Digital media outlets	290	20.1%
Social media	279	19.3%
Press	186	12.9%
Radio	166	11.5%

Source: own elaboration. "Media outlets (any category)" represents people who indicated any of the following: television, press, radio or digital media outlets.

**Table 7:** Sources cited in news content on radon (n_2_ = 1442).

Cited Source Regarding Radon	Frequency	
Do not remember	653	45.3%
Government	356	24.7%
NGO	241	16.7%
Spanish Nuclear Safety Council	215	14.9%
Political party	117	8.1%

Source: own elaboration.

**Table 8:** Information received through communication actions implemented by institutions or groups (n_2_ = 1442).

Origin of Communication Actions	%
None	58.2
Media outlets	19.5
European Union	10.3
Scientific institutions	8.4
Workplace	8.0
Friends and family	8.0
State government	7.6
Regional government	6.2
School	4.8
Spanish Nuclear Safety Council	4.6
Local government	4.6
Faculty	3.5
Others	0.1

Source: own elaboration.

## Data Availability

Data in this study will be shared on a reasonable request to the corresponding author and following the data management guidelines of this project.

## Supplementary Materials

The following supporting information can be downloaded at: https://www.mdpi.com/article/10.3390/ijerph21101302/s1. Table S1. Sample of digital news media and items analysed; Table S2. Variables of the content analysis sheet.
