# Supplementary material for: Radon Risk Communication through News Stories: A Multi-Perspective Approach
Source: Int J Environ Res Public Health. 2024 Sep 29;21(10):1302. doi: 10.3390/ijerph21101302 (PMC11506878; doi:10.3390/ijerph21101302)
Supplement: Supplementary file 1 [file ijerph-21-01302-s001.zip › ijerph-3140333-supplementary.pdf]

International Journal of
Environmental Research and Public Health

MDPI

Supplementary Material

Table S1. Sample of digital news media and items analysed.

Digital media	Type of media; Region	Period of analysis*	No. of stories analysed
La Voz de Galicia (https://www.lavozdegalicia.es)	Legacy; Galicia	2002 – 2022	169
Faro de Vigo (https://www.farodevigo.es)	Legacy; Galicia	2007 – 2022	55
Diario Hoy (https://www.hoy.es)	Legacy; Extremadura	2006 – 2022	42
La Región (https://www.laregion.es)	Legacy; Galicia	2007 – 2022	32
El Norte de Castilla (https://www.elnortedecastilla.es)	Legacy; Castilla y León	2006 – 2022	31
El Periódico de Extremadura (https://www.elperiodicoextremadura.com/)	Legacy; Extremadura	2007 – 2022	30
El Comercio (https://www.elcomercio.es)	Legacy; Asturias	2007 – 2022	30
Salamanca 24Horas (https://www.salamanca24horas.com)	Digital native; Castilla y León	2013 – 2022	26
Diario de León (https://www.diariodeleon.es)	Legacy; Castilla y León	2006 – 2022	23
Diario de Sevilla (https://www.diariodesevilla.es)	Legacy; Andalucía	2007 – 2022	22
La Nueva España (https://www.lne.es)	Legacy; Asturias	2009 – 2022	20
Aquí en la Sierra (https://aquienlasierra.es)	Digital native; Madrid	2017 – 2022	19
Galicia Confidencial (https://www.galiciaconfidencial.com)	Digital native; Galicia	2013 – 2022	15
iLeón (https://ileon.eldiario.es)	Digital native; Castilla y León	2016 – 2022	13
Diario Córdoba (https://www.diariocordoba.com)	Legacy; Andalucía	2011 – 2022	11
Región Digital (https://www.regiondigital.com)	Digital native; Extremadura	2017 – 2022	10
La Gaceta de Salamanca (https://www.lagacetadesalamanca.es)	Legacy; Castilla y León	2011 – 2022	10
LaCerca.com (https://www.lacerca.com)	Digital native; Castilla-La Mancha	2010 – 2022	9
El Adelantado (https://www.eladelantado.com)	Legacy; Castilla y León	2018 – 2022	4
La Tribuna de Ciudad Real (https://www.latribunadeciudadreal.es)	Legacy; Castilla-La Mancha	2021 – 2022	4
Lanza Digital (https://www.lanzadigital.com)	Digital native; Castilla-La Mancha	2010 – 2022	3
La Plaza Información (http://www.laplazainformacion.com)	Digital native; Andalucía	N/A	0
InfoGuadiato (https://infoguadiato.com)	Digital native; Andalucía	N/A	0

Source: own elaboration. *Period differs for each media outlet because the starting year corresponds to the date of publication of the oldest retrieved news item.

Table S2. Variables of the content analysis sheet.

**Identification data**	News outlet; publication date; headline; authorship; thematic section; URL
**Main topic**	Health and prevention; housing and urban planning; research; policy and regulation; environment; other.
**News values**	Novelty; relevance; conflict; impact; personalization.
**Magnitudes of risk**	Spatial exposure; exposure and risk; nature of effects; sources of gas; reversibility of effects; reliance on institutions.
**Sources of information**	Number of sources; typology (public administration, political parties, research institutions, business, experts, associations, citizens); identification; proximity of sources.
**Geographical scope**	International; national; region; local; places mentioned.

Source: own elaboration.
